# Interleukin-15 (IL-15) Strongly Correlates with Increasing HIV-1 Viremia and Markers of Inflammation

**DOI:** 10.1371/journal.pone.0167091

**Published:** 2016-11-23

**Authors:** Sanjay Swaminathan, Ju Qiu, Adam W. Rupert, Zonghui Hu, Jeanette Higgins, Robin L. Dewar, Randy Stevens, Catherine A. Rehm, Julia A. Metcalf, Brad T. Sherman, Michael W. Baseler, H. Clifford Lane, Tomozumi Imamichi

**Affiliations:** 1 Applied and Developmental Research Directorate, Leidos Biomedical Research, Inc., Frederick National Laboratory for Cancer Research, Frederick, MD, 21702, United States of America; 2 Department of Clinical Immunology, Western Sydney Local Health District, Sydney, Australia; 3 Sydney Medical School, University of Sydney, Sydney, Australia; 4 School of Medicine, Western Sydney University, Sydney, Australia; 5 Biostatistics Research Branch, National Institute of Allergy and Infectious Diseases (NIAID), National Institutes of Health (NIH), Bethesda, MD, 20892, United States of America; 6 Laboratory of Immunoregulation, National Institute of Allergy and Infectious Diseases, National Institutes of Health, Bethesda, MD, 20892, United States of America; 7 Division of Clinical Research, NIAID, NIH, Bethesda, MD, 20892, United States of America; University of South Carolina School of Medicine, UNITED STATES

## Abstract

**Objective:**

IL-15 has been postulated to play an important role in HIV-1 infection, yet there are conflicting reports regarding its expression levels in these patients. We sought to measure the level of IL-15 in a large, well characterised cohort of HIV-1 infected patients and correlate this with well known markers of inflammation, including CRP, D-dimer, sCD163 and sCD14.

**Design and Methods:**

IL-15 levels were measured in 501 people (460 patients with HIV-1 infection and 41 uninfected controls). The HIV-1 infected patients were divided into 4 groups based on viral load: <50 copies/ml, 51–10,000 copies/ml, 10,001–100,000 copies/ml and >100,000 copies/ml. The Mann Whitney test (non-parametric) was used to identify significant relationships between different patient groups.

**Results:**

IL-15 levels were significantly higher in patients with viral loads >100,000 copies/ml (3.02 ± 1.53 pg/ml) compared to both uninfected controls (1.69 ± 0.37 pg/ml, p<0.001) or patients with a viral load <50 copies/ml (1.59 ± 0.40 pg/ml (p<0.001). There was a significant correlation between HIV-1 viremia and IL-15 levels (Spearman r = 0.54, p<0.001) and between CD4+ T cell counts and IL-15 levels (Spearman r = -0.56, p<0.001).

**Conclusions:**

IL-15 levels are significantly elevated in HIV-1 infected patients with viral loads >100,000 copies/ml compared to uninfected controls, with a significant direct correlation noted between IL-15 and HIV-1 viremia and an inverse correlation between IL-15 levels and CD4+ T cell counts. These data support a potential role for IL-15 in the pathogenesis of HIV-associated immune activation.

## Introduction

Interleukin-15 (IL-15), along with IL-2, IL-4, IL-7, IL-9 and IL-21, belongs to a four α-helix bundle family of cytokines that all utilize the common-gamma chain of the IL-receptor. It is a pleiotropic cytokine with important roles in both the innate and adaptive immune systems [1]. IL-15 has been demonstrated to expand and differentiate Natural Killer (NK) cells, as well as to play an important role in macrophage maturation. Other roles of IL-15 within the innate immune system include maturation of NK-T cells and intraepithelial lymphocytes as well as effects on a number of other cell types including dendritic cells, neutrophils, eosinophils, mast cells and various B and T cell subsets [2].

One of the better studied aspects of IL-15 has been its role as a survival factor for memory T cells, particularly memory CD8+ T cells. IL-15 is thought to promote proliferation of memory CD8+ T cells. A number of studies over the years have suggested that IL-15 may also play an important role in patients with HIV-1 infection [3, 4]. IL-15 has also shown to increase the production of γ-IFN, CCL4 and CCL5 in NK cells from HIV patients both on and off anti-retroviral (ART) therapy [5]. In addition, IL-15 has been touted as a potential therapeutic cytokine because of its potential to boost immune function by enhancing the survival of HIV specific CD8+ T cells [3, 6]. Previous work has shown that IL-15 is acutely elevated in seroconverters [7] but the results are not consistent [8]. In addition, some smaller studies have shown lower expression of IL-15 in HIV-1 infected individuals compared to control subjects [9]. Given the discrepancies in the literature regarding IL-15 levels in HIV-1 infection and desiring a better perspective on the potential role that IL-15 may play as a therapeutic cytokine, we sought to investigate the levels of IL-15 in a large well-characterised cohort of HIV-1 infected patients and compare it to biomarkers of inflammation. This is the largest study to date looking at the levels of IL-15 in HIV-1 patients stratified according to viral load and correlating this with other important biomarkers associated with HIV-1 infection.

## Methods

### Samples

The HIV-1 patients included in this study were enrolled in a number of National Institutes of Health Clinical Center protocols as listed in the acknowledgments. These protocols were approved by the National Institute of Allergy and Infectious Diseases (NIAID) Institutional Review Board, administered at the NIH Clinical Center in Bethesda, MD. All study participants provided informed written consent prior to blood being drawn. The HIV-1 positive patients were divided into four groups depending on HIV-1 viral load (<50 copies/ml, 51–10,000 copies/ml, 10,001–100,000 copies/ml and >100,000 copies/ml) and were comparable according to gender, age and combination antiretroviral therapy (cART) (Table 1). The specific ART treatment each of the patients were commenced on are listed in S1 Table. Additional information about the CD4 nadir, HIV-1 viral load, the duration of infection of HIV-1 and AIDS defining illnesses are listed in S2 Table.

**Table 1:** Demographics of patients used in study.

Group	<50 copies/ml	51–10,001 copies/ml	10,001–100,000 copies.ml	>100,000 copies/ml	Uninfected Controls	Totals
Number of patients^1^	115	108	116	118	44	501
No. of males^2^	84 (73.0%)	82 (75.9%)	92 (79.3%)	94 (79.9%)	25 (56.9%)	377 (75.2%)
No. of females^3^	31 (27.0%)	26 (24.1%)	24 (20.7%)	24 (20.3%)	19 (43.2%)	124 (24.8%)
Mean Age ± SD (years)	48.1 ± 9.3	42.9 ± 12.4	42.8 ± 10.5	39.2 ± 10.1	41.4 ± 11.2	43.1 ± 11.1
No. of patient on ART^4^	34 (30.0%)	26 (24.1%)	24 (20.7%)	31 (26.3%)	N/A	115
Hepatitis C	0	0	12	0	0	12
Hepatitis B	9	0	5	0	0	14
Mean Viral Load ± SD^5^	<50	2,066 ± 2,589	33,082 ± 23,699	423,987 ± 808,614	N/A	
Viral Load range	<50	53–9,9945	10,241–97,690	102,480–7,627,300	N/A	
Mean CD4+ T cell count ± SD^6^	765 ± 332	521 ± 261	343 ± 269	113 ± 165	Not measured	
CD4+ T cell count range^6^	143–1,814	36–1,370	1–1,234	0–841	Not measured	

1: All HIV-1 positive patients were enrolled in protocols approved by the National Institute NIAID Review Board administered at the NIH.

2–4: Numbers in parentheses indicate percentages of male or females in each group.

5: The HIV-1 viral load was measured using the bDNA or real-time PCR method. Data show means +SD (copies/ml).

6: total CD4+T cell count was performed by flow cytometer and absolute CD4 counts were obtained using a dual platform method using a Sysmex XT2000i hematology analyzer. Data indicate means +SD (cells/ml).

Of the 115 patients who were in the <50 copies/ml group, 53 were considered elite controllers with undetectable viral loads and off ART.

IL-15 and C-reactive protein (CRP) measurements were determined by running EDTA preserved plasma in duplicate on an electrochemiluminescence assay (Meso Scale Discovery, Rockville, MD). The thresholds of detection for CRP and IL-15 using the electrochemiluminescence assay were 0.008 ng/ml and 0.61 pg/mL, respectively. For the IL-15 Meso assay, antibodies were obtained from R&D Systems, Minneapolis, MN, USA. sCD14 levels (R&D Systems, Minneapolis, MN, USA) and sCD163 levels (Aviscera Bioscience Inc., Santa Clara, CA, USA) were determined by running EDTA preserved plasma in duplicates using ELISA kits. D-dimer levels were measured by running 200 μl of plasma with an Enzyme Linked Fluorescence Assay (bioMérieux, Marcy l'Etoile, France). The HIV-1 viral load was measured using the bDNA or real-time PCR method. Data are represented as means ± SD. Dual-platform CD4+ T cell counts were obtained by flow cytometer (BD FACS Canto II, BD Biosciences, San Jose, CA) and a hematology analyser (Sysmex XT2000i, Sysmex America, Lincolnshire, IL).

### Statistical analysis

Statistical analyses were performed using Prism 6 for Windows (Graph Pad) and comparisons between groups were performed using the Wilcoxon rank-sum statistic (Mann-Whitney). Correlations were calculated using the Spearman’s rank correlation. The multiple regression analyses were performed with SAS (version 9.3, SAS Institute).

## Results

**Fig 1:**
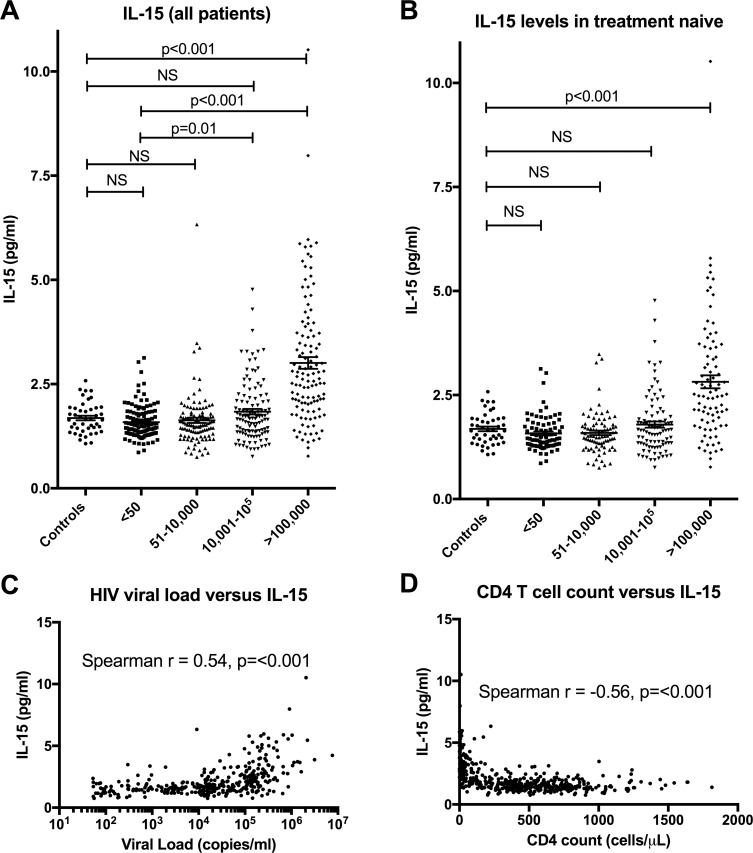
IL-15 levels in HIV-1 infected patients. HIV-1 infected patients were divided into 4 groups based on viral load and comparisons were made using the Mann-Whitney test (A)). In(B), comparisons were made for patients who were treatment naive. IL-15 levels were positively correlated with HIV-1 viral load (C) and negatively correlated with CD4+ T cell counts (D). Note that the viral load is graphed on a logarithmic scale (log 10) on the X-axis in (C).

IL-15 levels were measured and comparisons were made after stratifying HIV-1 positive patients into four groups, based on viral load, and comparing their levels with those of uninfected controls (Fig 1A). The mean level ± standard deviation (SD) of IL-15 was 1.69 ± 0.37 pg/ml in the uninfected controls, 1.59 ± 0.40 pg/ml in the <50 copies/ml group, 1.63 ± 0.66 pg/ml in the 51–10,000 copies/ml group, 1.84 ± 0.72 pg/ml in the 10,001–100,000 copies/ml group and 3.02 ± 1.53 pg/ml in the >100,000 copies/ml group. There was also a statistically significant increase in IL-15 levels noted between patients with <50 copies/ml and the 10,001–100,000 copies/ml group (p = 0.01) and between the <50 copies/ml group and the group with >100,000 copies/ml (p<0.0001) (Fig 1A).

As 23.1% of the HIV-1 positive patients were receiving ART, a separate analysis was made of treatment naïve patients. In this sub-analysis, the mean levels ± SD of IL-15 were 1.59 ± 0.50 pg/ml in the <50 copies/ml group, 1.57 ± 0.41 pg/ml, in the 51–10,000 copies/ml group, 1.79 ± 0.73 pg/ml in the 10,001–100,000 copies/ml group and 2.82 ± 1.46 pg/ml in the >100,000 copies/ml group. Patients with the highest viral loads (>100,000 copies/ml) had significantly higher levels compared to uninfected controls (2.82 vs. 1.70; p<0.001) (Fig 1B). A separate analysis was conducted for patients who were on ART and this showed the same trends as per the overall analysis with those with viral loads > 100,000 copies/ml having the highest levels of IL-15 (S1 Fig).

To investigate if there was a link between IL-15 and HIV-1 viral load, we used patient samples that had a specific viral load. We therefore excluded patients with viral loads <50 copies/ml, and analysed the remaining 342 patients (Fig 1C). The Spearman rank correlation between HIV-1 RNA and IL-15 levels was 0.54 (p = <0.001), demonstrating a strong positive correlation between these two parameters. We then looked at the relationship between IL-15 levels and CD4+ T cell counts in all HIV-1 positive patients in this study (457 patients) and found a negative correlation between IL-15 levels and CD4+ T cell counts (Spearman r = -0.56, p<0.001) (Fig 1D).

**Fig 2:**
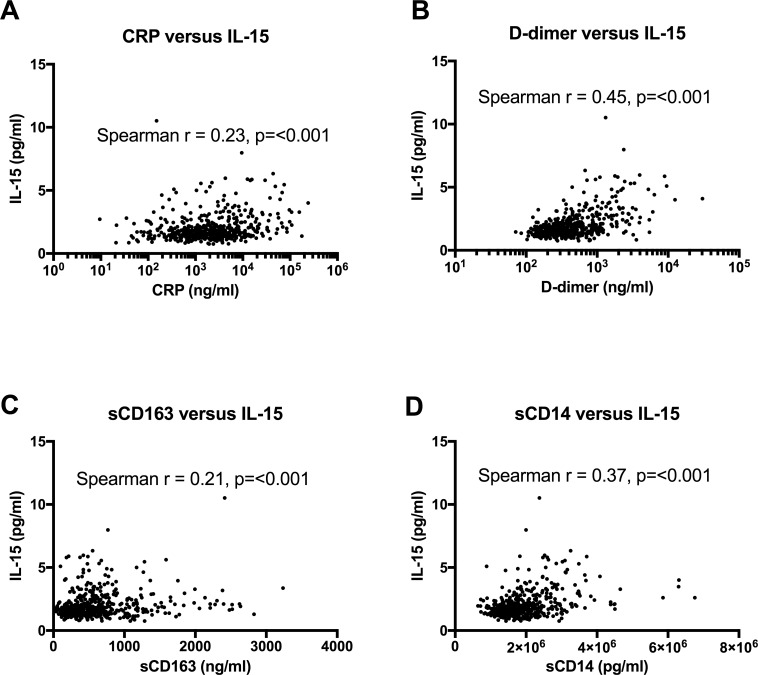
IL-15 levels were significantly correlated against markers of inflammation and coagulation. IL-15 levels showed significant correlation with CRP (A), D-dimer (B), sCD163 (C) and sCD14 (D).

There is increasing evidence to suggest that immune activation in HIV-1 infection may play an important role in predicting disease progression. We sought to see if there was a correlation between IL-15 and four markers of immune activation in HIV, namely C-reactive protein (CRP), D-dimer, sCD163 and sCD14. A significant correlation was noted with all 4 markers (Fig 2). Given the fact that a number of the variables measured in this study may be linked, a multiple regression analysis was performed. The regression model is significant based on the F value and associated p-value. The significant parameters showed significant correlations between IL-15 versus viral load, CD4+ T cell count, D-dimer and sCD14. CRP and sCD163 have weaker correlation with IL-15 levels but do not have significant p values using the multivariate analysis. S3 Table show the multivariate analysis table for those on ART whilst S4 Table shows the multivariate analysis for those who were ART naïve.

## Discussion

IL-15 has been proposed to potentially play a role in HIV-1 infection as either a vaccine adjuvant or as a therapeutic cytokine. However, IL-15 therapy has even been postulated to play a role in disease progression and to increase viral load in non-primate models infected with SIV [10]. Despite this, surprisingly little is known regarding the levels of expression of IL-15 in patients chronically infected with HIV-1. Early reports into the expression of IL-15 in primary infection show conflicting results, with both high and low results being reported [11].

Prior to this study, only small numbers of patients had been studied specifically looking at IL-15 production. Those studies concluded that IL-15 was decreased in chronic HIV-1 infection. Our findings in a larger cohort paint a different picture with clear evidence of increased levels of IL-15 as the viral load increases or as the CD4+ T cell count decreases. These increases in IL-15 were closely correlated with markers of inflammation and coagulation and appear to be part of the overall immune activation seen in the context of HIV-1 infection. In addition to a larger sample size, a difference with our study compared to earlier reports is the use of a robotic platform for measurement that minimizes operator variability.

One particular hallmark of chronic viral infections, including HIV-1, is an activated, sometimes referred to as “exhausted” phenotype of memory CD8+ T cells. Murine models of chronic infection show that CD8+ T cells from these animals express low levels of CD122 (the β chain of the IL-2 and IL-15 receptor) and respond poorly to these cytokines [12]. One may speculate that in chronic HIV-1 infection, CD4 and CD8+ T cells that are poorly responsive to normal physiological and homeostatic pathways involving IL-15, may need higher circulating levels of IL-15 to remain functional. Further work longitudinally following the changes in cell surface markers in memory CD4 and CD8+ T cells in chronic HIV infection and correlating these with IL-15 expression levels may provide more information about possible links.

IL-15 is produced by a number of cells including monocytes, macrophages and dendritic cells. We sought to see if there was a correlation of IL-15 in HIV-1 infection with other markers of monocyte activation, including sCD14 and sCD163 and indeed found that both of these parameters were significantly correlated with IL-15 expression. One interesting difference between expression levels of IL-15 and markers of monocyte activation used in this study is that IL-15 levels are trending lower in patients with undetectable viral loads, whilst these same patients have significantly higher levels of both sCD14 and sCD163 (data not shown) in patients who are virally suppressed. This suggests that IL-15 may be a better marker of disease progression than either sCD14 or sCD163, particularly at lower levels of viremia, although prospective studies would need to be conducted to answer this question definitively.

It has been suggested that IL-15, as well as other cytokines that are expressed in high levels in acute infection, may play a role in establishing the HIV reservoir [13]. It would have been interesting to note whether there was a correlation between total and integrated DNA in ART treated patients and IL-15 levels in our cohort to test this hypothesis.

The most important finding from this study is that IL-15 levels are not decreased in the setting of HV-1 infection but rather show a significant positive correlation with HIV-1 viremia, particularly at viral loads>100,000 copies/ml. These increases correlate with well-known markers of inflammation and coagulation that have been used as predictors for clinical progression of HIV-1 infection.

## Supporting Information

S1 Fig Correlation between IL-15 level and HIV viral load in patients treated with ART. HIV-1 infected patients with ART were divided into 4 groups based on viral load and comparisons were made with IL-15 level using the Mann-Whitney test.
(TIFF)

S1 Table ART treatment regimen for each patient. PO: oral administration, SQ: subcutaneous injection, QID: once a day, TID: three times a day, BID: twice a day, QHS: at every bed time.
(XLSX)

S2 Table Information of each patient’s viral load, CD4 counts and AIDS defining illness. (XLSX)

S3 Table The multivariate analysis for those who were on ART. (DOCX)

S4 Table The multivariate analysis for those who were ART naïve. (DOCX)

## References

[1] Ma A, Koka R, Burkett P. Diverse functions of IL-2, IL-15, and IL-7 in lymphoid homeostasis. Annual review of immunology. 2006;24:657–79. Epub 2006/03/23. 10.1146/annurev.immunol.24.021605.090727 . 16551262

[2] Verbist KC, Klonowski KD. Functions of IL-15 in anti-viral immunity: multiplicity and variety. Cytokine. 2012;59(3):467–78. Epub 2012/06/19. 10.1016/j.cyto.2012.05.020 22704694 PMC3422395

[3] Chehimi J, Marshall JD, Salvucci O, Frank I, Chehimi S, Kawecki S, et al IL-15 enhances immune functions during HIV infection. Journal of immunology. 1997;158(12):5978–87. Epub 1997/06/15. . 9190952

[4] Kanai T, Thomas EK, Yasutomi Y, Letvin NL. IL-15 stimulates the expansion of AIDS virus-specific CTL. Journal of immunology. 1996;157(8):3681–7. Epub 1996/10/15. . 8871670

[5] d'Ettorre G, Forcina G, Lichtner M, Mengoni F, D'Agostino C, Massetti AP, et al Interleukin-15 in HIV infection: immunological and virological interactions in antiretroviral-naive and -treated patients. Aids. 2002;16(2):181–8. Epub 2002/01/25. . 11807301 10.1097/00002030-200201250-00006

[6] Mueller YM, Bojczuk PM, Halstead ES, Kim AH, Witek J, Altman JD, et al IL-15 enhances survival and function of HIV-specific CD8+ T cells. Blood. 2003;101(3):1024–9. Epub 2002/10/24. 10.1182/blood-2002-07-1957 . 12393488

[7] Stacey AR, Norris PJ, Qin L, Haygreen EA, Taylor E, Heitman J, et al Induction of a striking systemic cytokine cascade prior to peak viremia in acute human immunodeficiency virus type 1 infection, in contrast to more modest and delayed responses in acute hepatitis B and C virus infections. Journal of virology. 2009;83(8):3719–33. Epub 2009/01/30. 10.1128/JVI.01844-08 19176632 PMC2663284

[8] Boulassel MR, Young M, Routy JP, Sekaly RP, Tremblay C, Rouleau D. Circulating levels of IL-7 but not IL-15, IGF-1, and TGF-beta are elevated during primary HIV-1 infection. HIV clinical trials. 2004;5(5):357–9. Epub 2004/11/25. 10.1310/M0CV-R6BX-A9DP-JJV0 . 15562373

[9] Ahmad R, Sindhu ST, Toma E, Morisset R, Ahmad A. Studies on the production of IL-15 in HIV-infected/AIDS patients. Journal of clinical immunology. 2003;23(2):81–90. Epub 2003/05/22. . 12757260 10.1023/a:1022568626500

[10] Lugli E, Mueller YM, Lewis MG, Villinger F, Katsikis PD, Roederer M. IL-15 delays suppression and fails to promote immune reconstitution in virally suppressed chronically SIV-infected macaques. Blood. 2011;118(9):2520–9. 10.1182/blood-2011-05-351155 21757617 PMC3167360

[11] Mastroianni CM, d'Ettorre G, Forcina G, Vullo V. Teaching tired T cells to fight HIV: time to test IL-15 for immunotherapy? Trends Immunol. 2004;25(3):121–5. 10.1016/j.it.2004.01.002 . 15036038

[12] Wherry EJ. T cell exhaustion. Nature immunology. 2011;12(6):492–9. Epub 2011/07/09. . 21739672 10.1038/ni.2035

[13] Vandergeeten C, Fromentin R, Chomont N. The role of cytokines in the establishment, persistence and eradication of the HIV reservoir. Cytokine Growth Factor Rev. 2012;23(4–5):143–9. 10.1016/j.cytogfr.2012.05.001 22743037 PMC3767481

