# Supplementary figures and images for: Interleukin-15 (IL-15) Strongly Correlates with Increasing HIV-1 Viremia and Markers of Inflammation
Source: PLoS One. 2016 Nov 23;11(11):e0167091. doi: 10.1371/journal.pone.0167091 (PMC5120855; doi:10.1371/journal.pone.0167091)

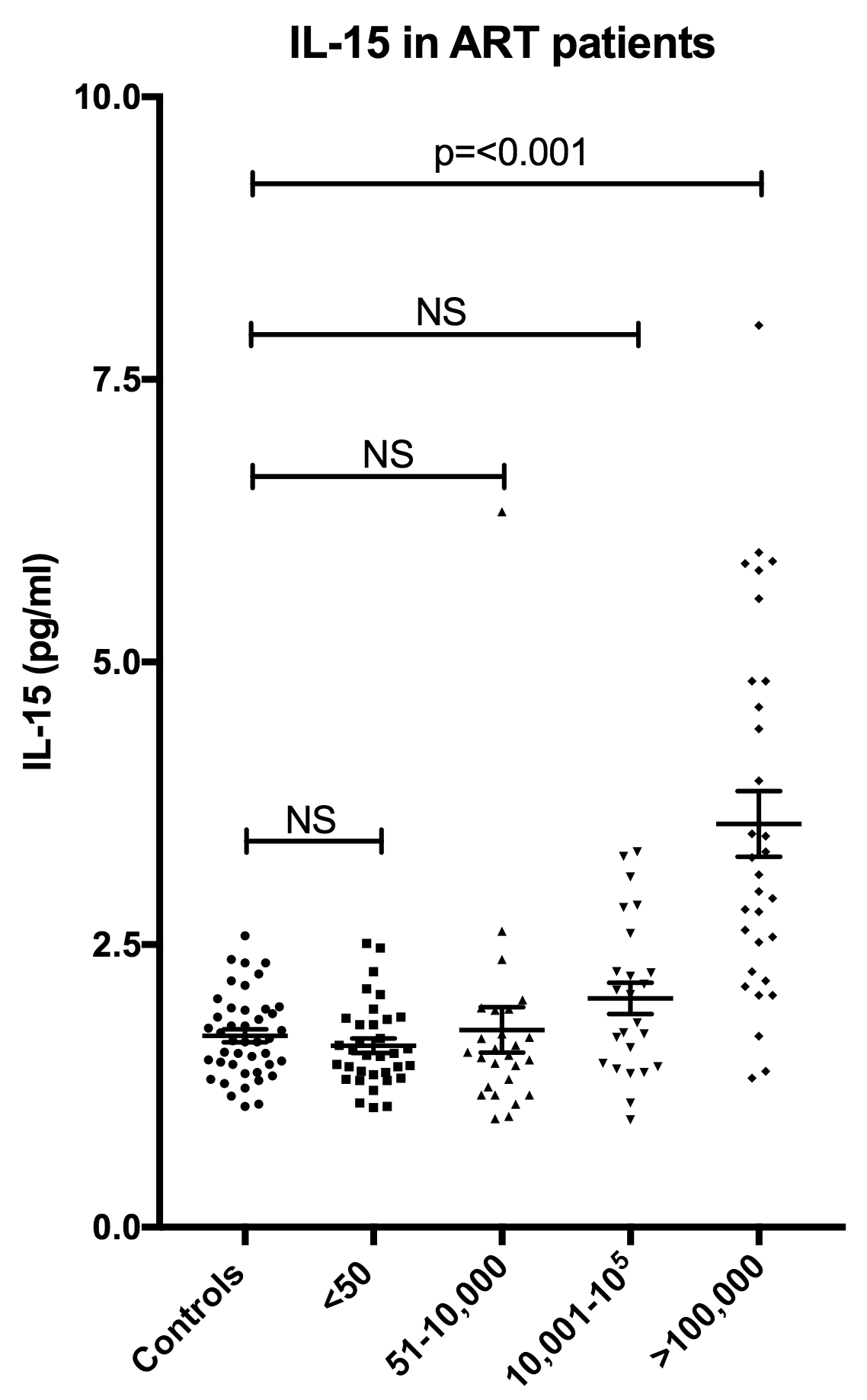

Supplement: S1 Fig — HIV-1 infected patients with ART were divided into 4 groups based on viral load and comparisons were made with IL-15 level using the Mann-Whitney test. (TIFF) [file pone.0167091.s001.tiff]

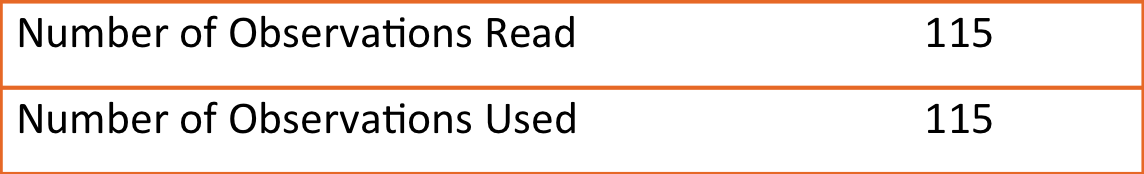

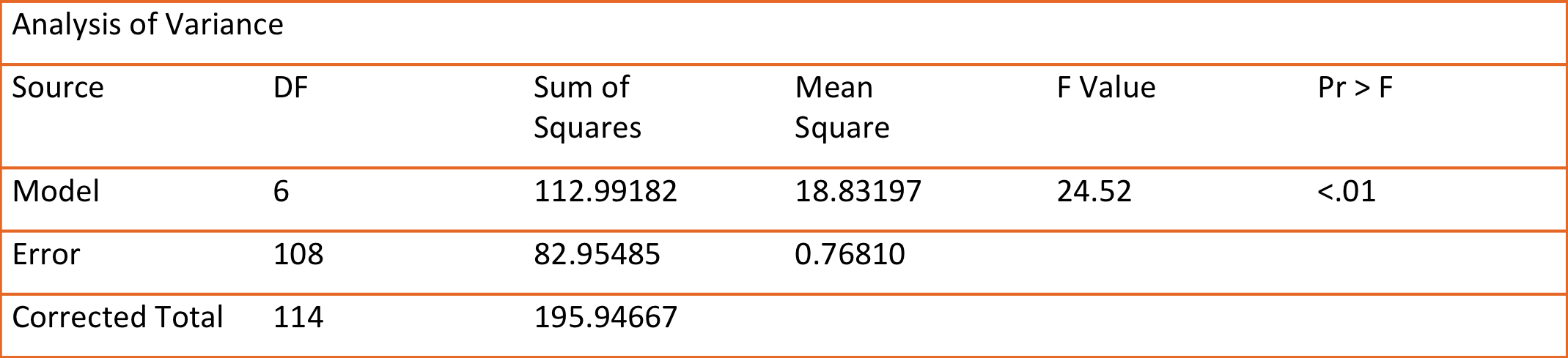

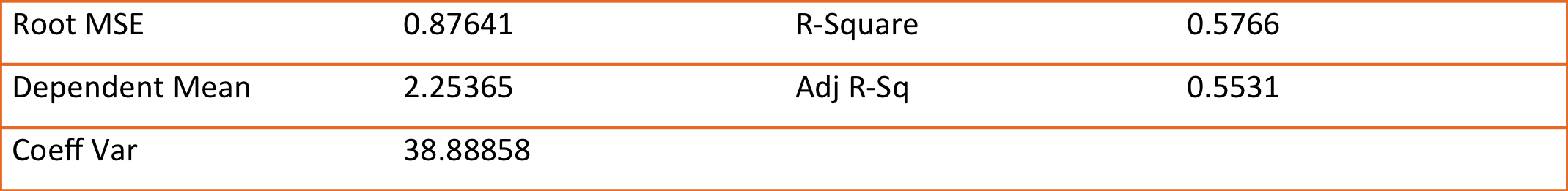

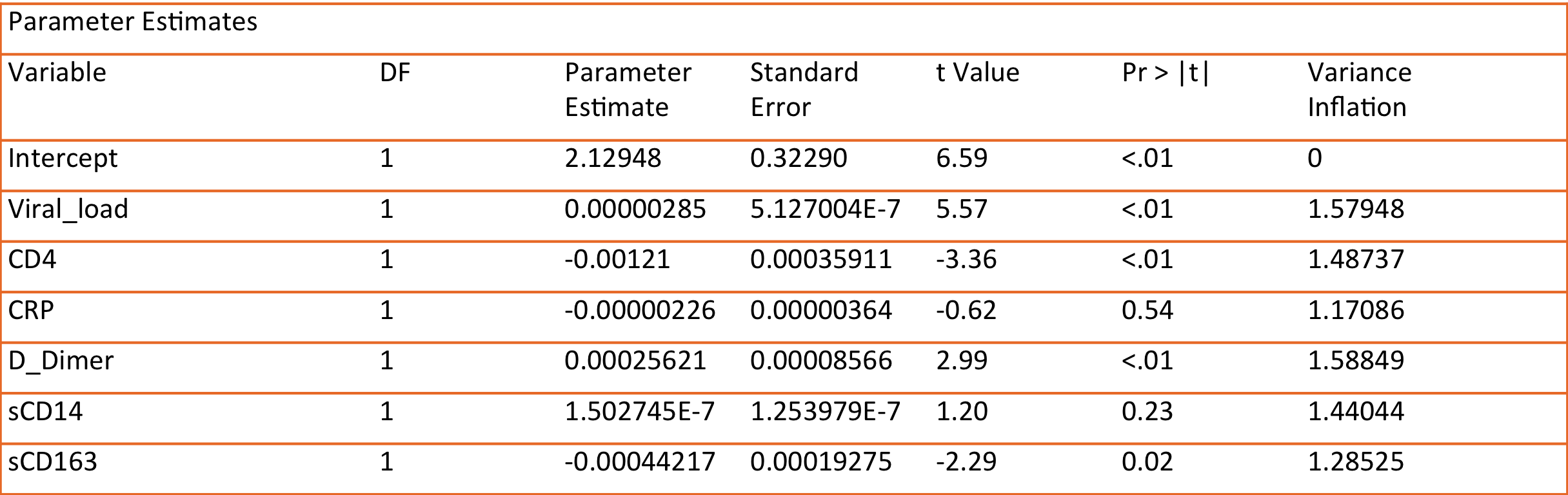


S3 Table: Multivariate analysis of patients on ART

Supplement: S3 Table — (DOCX) [file pone.0167091.s004.docx]

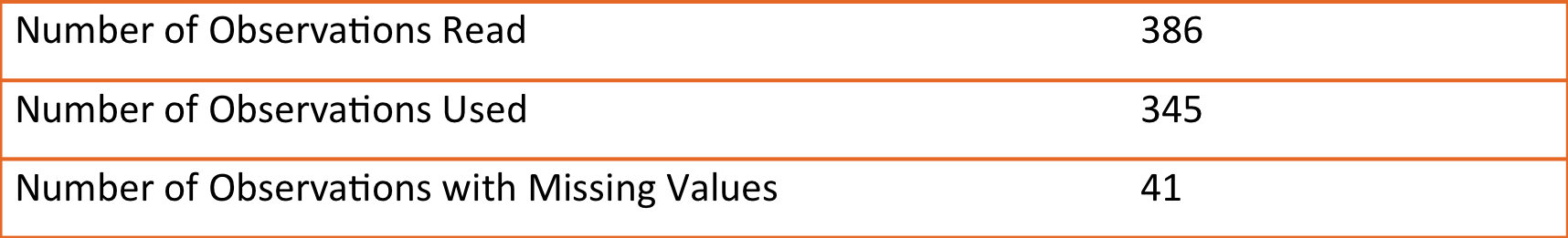

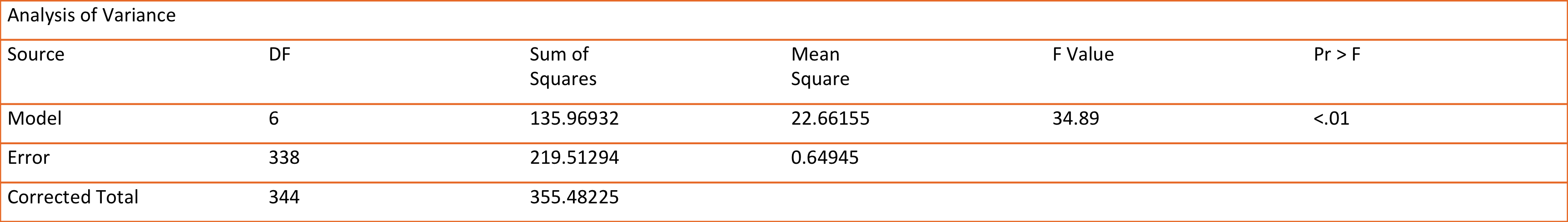

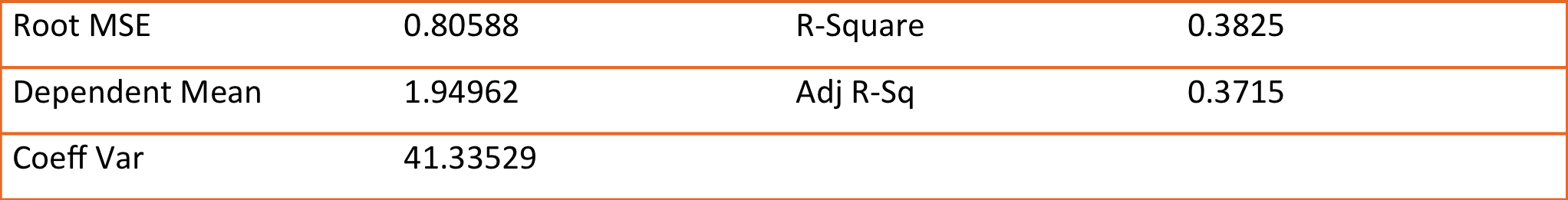

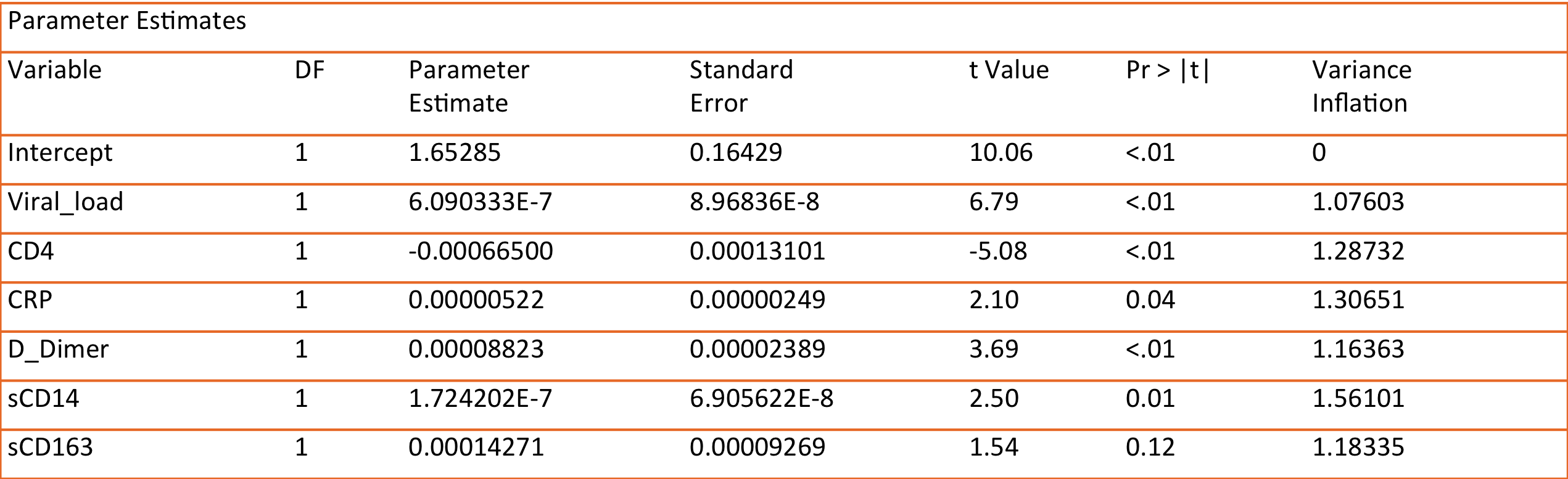


S4 Table: Multivariate analysis on ART naïve patients

Supplement: S4 Table — (DOCX) [file pone.0167091.s005.docx]
